# Phylogeny and Comparative Analysis for the Plastid Genomes of Five *Tulipa* (Liliaceae)

**DOI:** 10.1155/2021/6648429

**Published:** 2021-06-18

**Authors:** Juan Li, Megan Price, Dan-Mei Su, Zhen Zhang, Yan Yu, Deng-Feng Xie, Song-Dong Zhou, Xing-Jin He, Xin-Fen Gao

**Affiliations:** ^1^Key Laboratory of Bio-Resources and Eco-Environment of Ministry of Education, College of Life Sciences, Sichuan University, Chengdu, 610065 Sichuan, China; ^2^Sichuan Key Laboratory of Conservation Biology on Endangered Wildlife, College of Life Sciences, Sichuan University, Chengdu, 610065 Sichuan, China; ^3^CAS Key Laboratory of Mountain Ecological Restoration and Bioresource Utilization & Ecological Restoration and Biodiversity Conservation Key Laboratory of Sichuan Province, Chengdu Institute of Biology, Chinese Academy of Sciences, Chengdu, 610041 Sichuan, China

## Abstract

Species of *Tulipa* (Liliaceae) are of great horticultural importance and are distributed across Europe, North Africa, and Asia. The Tien Shan Mountain is one of the primary diversity centres of *Tulipa*, but the molecular studies of *Tulipa* species from this location are lacking. In our study, we assembled four *Tulipa* plastid genomes from the Tien Shan Mountains, *T. altaica*, *T. iliensis*, *T. patens*, and *T. thianschanica*, combined with the plastid genome of *T. sylvestris* to compare against other Liliaceae plastid genomes. We focussed on the species diversity and evolution of their plastid genomes. The five *Tulipa* plastid genomes proved highly similar in overall size (151,691–152,088 bp), structure, gene order, and content. With comparative analysis, we chose 7 mononucleotide SSRs from the *Tulipa* species that could be used in further population studies. Phylogenetic analyses based on 24 plastid genomes robustly supported the monophyly of *Tulipa* and the sister relationship between *Tulipa* and *Amana*, *Erythronium*. *T. iliensis*, *T. thianschanica*, and *T. altaica* were clustered together, and *T. patens* was clustered with *T. sylvestris*, with our results clearly demonstrating the relationships between these five *Tulipa* species. Our results provide a more comprehensive understanding of the phylogenomics and comparative genomics of *Tulipa*.

## 1. Introduction

Plastid, a unique semiautonomous organelle of green plants, serves as a protagonist in photosynthesis and carbon fixation and provides essential energy for plants [1, 2]. Plastid DNA has been widely used in evolutionary biology analyses because of its uniparental inheritance, relatively stable genome structure, and gene content [3–7]. A plastid genome has a quadripartite circular structure consisting of two copies of inverted repeat (IR) regions, a large single copy (LSC) region, and a small copy region (SCR) in most seed plants [8–10]. Comparative genomics of whole plastid genomes has been used to generate genetic markers for molecular identification [11, 12]. Plastid genomic data provide new and robust insights into phylogenetic analysis and genetic variation detection of plants [13].

Tulips (*Tulipa* L.) are famous ornamental and cut flowers due to their beautiful and colorful corolla [14–16]. *Tulipa* is a member of Liliaceae sensu APG IV [17], subfamily Lilioideae, tribe Tulipeae Kostel [18, 19]; the tribe Tulipeae including *Gagea* Salisbury, *Amana* Honda, *Erythronium* L., and *Tulipa* [20] is sister to the tribe Lilieae [21]. The close relationship between *Gagea* and *Tulipa*, *Amana*, and *Erythronium* is generally accepted; however, the phylogenetic relationships among *Tulipa*, *Amana*, and *Erythronium* have remained controversial due to inconsistencies across studies and lack of strong support [22, 23]. *Amana* used to be treated as a group in *Tulipa* [24–26] but is now generally accepted as a separate genus [27–29]. Several studies have supported a sister relationship between *Tulipa* and *Amana* [30, 31], whereas others clustered *Tulipa* and *Erythronium* together [22, 29]. A close relationship between *Amana* and *Erythronium* has been suggested; however, the *Tulipa* data used in these studies was limited [31–33].

*Tulipa* includes more than 100 species [34, 35] and are distributed across the temperate regions of Europe, North Africa, and Asia. The Tien Shan, Pamir-Alay, and Caucasus Mountains are considered the primary diversity centres of *Tulipa* [36, 37]. Thirteen species of *Tulipa* are recorded and described in China, and 11 species are distributed in the Xinjiang Uygur Autonomous Region [37], which is home to the Tien Shan Mountain. Several studies have utilised a few plastid regions and internal transcribed spacer makers to analyse the phylogeny and evolution of *Tulipa* in the Middle East [38, 39] and Europe [29]. However, the phylogeny and evolution of *Tulipa* at Tien Shan Mountain are limited. Two *Tulipa* plastid genomes have been published [40, 41], but genomics analysis for *Tulipa* is lacking. Therefore, comprehensive studies including the specimens from Tien Shan Mountain and accurate analysis of the plastid genome are required to enable further *Tulipa* molecular studies.

We collected four *Tulipa* species from Tien Shan Mountain and reported the complete plastid genome sequences of these four *Tulipa* species to address gaps in *Tulipa* phylogenetic research. Combining previously reported plastid genomes of *T. sylvestris* and other Liliaceae species, we performed comparative genomics and phylogenetic analyses. We aimed to resolve the phylogenetic relationships between five *Tulipa* species and other genera and characterize and compare the plastid genomes of *Tulipa* species to detect the genetic variation. Our conclusion will contribute to an understanding of *Tulipa* plastid phylogenomics and provide genetic resources for tulip research.

## 2. Materials and Methods

### 2.1. Plant Materials, DNA Extraction, and Sequencing

Fresh leaves of four *Tulipa* species, *T. altaica*, *T. iliensis*, *T. patens*, and *T. thianschanica*, were collected from Yuming county (Xinjiang Uygur Autonomous Region, China) and dried with silica gel, then stored at -80°C. Total genomic DNA was extracted from leaf material with a modified CTAB method [42] and then sequenced on an Illumina Novaseq2500 sequencer (Illumina, San Diego, CA, USA) by Biomarker Technologies, Inc. (Beijing, China).

### 2.2. Plastid Genome Assembly, Annotation, and Analysis

The plastid genomes were assembled using raw data by NOVOPlasty 2.7.2 [43], and the plastid genome of *T. sylvestris* (MT261172) was selected for seed input and the reference sequence. Genome annotation and IR region search were processed by PGA [44]. Geneious R11 [45] was used on manual modifications to accurately confirm the start and stop codons and the exon-intron boundaries of genes based on comparison with other Liliaceae plastid genomes. The circular plastid genome map of *Tulipa* was drawn by the OGDRAW1 program [46]. The total GC content and GC content of each region (IR, LSC, SSC) were analysed by the program Geneious R11.

### 2.3. Contraction and Expansion of IRs and SSRs

Four plastid genomes of the tribe Tulipeae, *Amana edulis* (NC034707), *Erythronium japonicum* (MT261155), *Erythronium sibiricum* (NC035681), and *Gagea triflora* (MT261157), were downloaded from the GenBank for comparative analysis with five *Tulipa* species.

The IR/SC borders with full annotations were compared between the five *Tulipa* species and with the other four tribe Tulipeae species using the program IRscope (https://irscope.shinyapps.io/irapp/) [47]. Simple sequence repeats (SSRs) were detected using Perl script MISA [48] with the following minimum number (threshold) settings: 10, 5, 4, 3, 3, and 3 repeat units for mono-, di-, tri-, tetra-, penta-, and hexanucleotide SSRs, respectively.

### 2.4. Phylogenetic Analyses

To reconstruct phylogenetic relationships between the five *Tulipa* species and other Liliaceae species, a total of 20 plastid genome sequences were downloaded from GenBank, and *Smilax china* (Smilacaceae, HM536959) was selected as an outgroup. The alignment of 24 plastid genome sequences was generated by MAFFT v7.402 [49] with the default parameters set. The best-fit model selected by ModelFinder was GTR+G. A maximum likelihood (ML) tree was constructed using RAxML 8.0 [50] with 1000 bootstrap replicates.

### 2.5. Codon Usage Analysis and SNP Analyses

All 84 protein-coding sequences extracted from nine plastid genomes were used to analyse codon usage, which was undertaken with the CodonW v1.4.2 program (J. Peden, http://codonw.sourceforge.net). The plastid genome sequences of nine tribe Tulipeae species were used for SNP analyses. The alignment of all plastid genome sequences was generated by MAFFT v7.402. SNP analysis was conducted to determine the nucleotide diversity of the plastid genomes using DnaSP v5, with the following parameters: 200 bp of step size and 600 bp window length [51]. Results of the SNP analysis were illustrated using TBtools v1.087 software [52].

## 3. Results and Discussion

### 3.1. The Plastid Genomes of Five Tulipa Species

**Figure 1:**
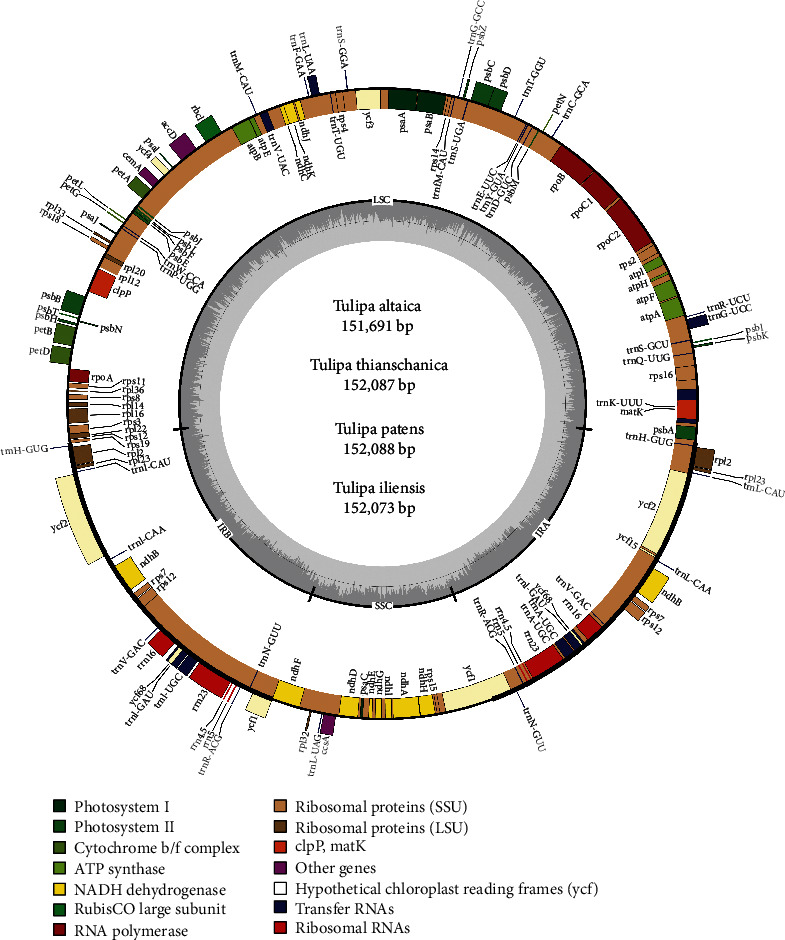
Plastid genome map of *T. altaica*, *T. iliensis*, *T. patens*, *T. sylvestris*, and *T. thianschanica*.

The total plastid genome sizes of the five *Tulipa* species ranged from 151691 bp (*T. altaica*) to 152088 bp (*T. patens*). All five plastid genomes showed the typical quadripartite structure (Figure 1) and, like other angiosperms, consisted of a pair of IR regions. The G+C content of the five species in whole genomes (36.6-36.7) and LSC (34.5-34.6), SSC (30.0-30.2) was nearly identical but in the IR regions was higher (42.0%). Details of genome features are given in Table 1. The annotated genome sequences of *T. altaica*, *T. iliensis*, *T. patens*, and *T. thianschanica* were deposited in the GenBank under the accession numbers MW077741, MW077740, MW077739, and MW077738, respectively (Table 1).

The plastid genomes of five *Tulipa* species contained 134 genes. Of these 134 genes, 112 genes were nonredundant including 78 protein-coding genes, 4 ribosomal RNA (rRNA) genes, and 30 transfer RNA (tRNA) genes, and four genes were pseudogenes (Table 2). The 134 genes had 18 duplicated genes located in the IR region, including six coding genes (*ndh*B, *rpl*2, *rpl*23, *rps*7, *rps*12, and *ycf*2), four rRNA genes (*rrn*4.5, *rrn*5, *rrn*16, and *rrn*23), and eight tRNA genes (*trn*A-UGC, *trn*H-GUG, *trn*I-CAU, *trn*I-GAU, *trn*L-CAA, *trn*N-GUU, *trn*R-ACG, and *trn*V-GAC).

Four pseudogenes (*ycf*1, *rps*19, and two *ycf*68) were found in the five plastid genomes (Table 2). The *rps*19 and *ycf*1 genes were located in the boundary area of the IR regions, and their protein-coding ability was lost due to partial gene duplication [6, 10, 40, 53]. Whether *ycf*68 and *ycf*15 genes lost abilities or occur as pseudogenes has already been discussed in several studies [40, 53, 54]. In this study, the *ycf*15 gene was not annotated due to its short length. The *inf*A gene, which codes for translation initiation factor 1, was lost in all five *Tulipa* plastid genomes because of a missing base. The deletion of the *inf*A gene also occurred in *Amana* and *Erythronium* [40], which were sister relationships with *Tulipa*, and many other seed plants, such as *Smilax* (Smilacaceae) [40] and *Alstroemeria* (Alstroemeriaceae) [33].

### 3.2. Inverted Repeats Contraction, Expansion, and SSR Analysis

**Figure 2:**
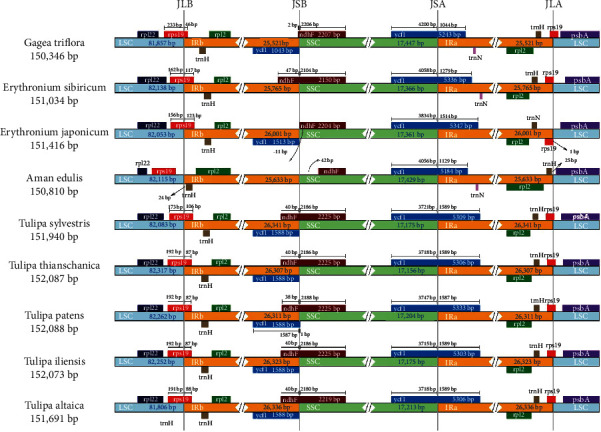
Comparison of the LSC, IR, and SSC junction positions among nine tribe Tulipeae plastid genomes. JLB: junction of LSC and IRb; JSB: junction of SSC and IRb; JSA: junction of SSC and IRa; JLA: junction of LSC and IRa.

The IR/SC boundary regions of the *Tulipa* plastid genomes were compared to the closely related plastid genomes, *Amana*, *Erythronium*, and *Gagea*. Typically, the lengths of IR regions are different among various plant species [53], while the lengths of *Tulipa* plastid genome IR regions were similar (26307 bp-26341 bp) but larger than *Amana* (25633 bp), *Erythronium* (25765 bp and 26001 bp), and *Gagea* (25521 bp) plastid genome IR regions (Figure 2). Furthermore, the expansion and contraction at the IR regions were the primary cause of size variation in plastid genomes and played an important role in the evolution of the genome [55–57]. After comparing the location and adjacent genes of IR regions between nine plastid genomes, we found that the gene number and order were conserved, but some distinct differences existed at the boundaries (Figure 2). The boundary of the LSC and IRb regions was located at *rps*19 genes in eight plastid genomes and positioned at the noncoding region between *rps*19 and *rpl*2 genes in the *Amana edulis* plastid genome. The *ndh*F and *ycf*1 genes traversed the regions of IRb/SSC and SSC/IRa, with 38/40 bp of the *ndh*F gene and 1587 bp/1589 bp of the *ycf*1 gene located at the IR region in the *Tulipa* plastid genome. In general, the length and structure of IR regions were similar in *Tulipa* genomes but showed obvious differences with other tribe Tulipeae genomes.

**Figure 3:**
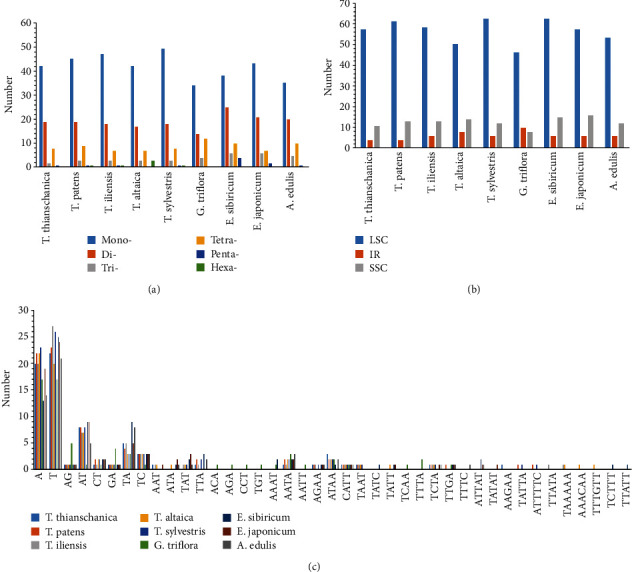
Analyses of simple sequence repeats (SSRs) in nine tribe Tulipeae plastid genomes: (a) numbers of different repeat types; (b) frequency of repeat types in LSC, SSC, and IR regions; (c) numbers of identified SSR motifs.

Simple sequence repeats (SSRs) in the plastid genomes are suitable molecular makers and have been widely used in evolutionary and ecological studies due to their high variation [58–64]. Given that SSRs have high polymorphism at the species level and commonly show intraspecific variation, SSRs were used as important molecular markers to reconstruct phylogenetic relationships [65–67]. The results of SSR analysis of *Tulipa* and its close relatives are shown in Figure 3 and Table S1-S2. There were six categories of SSRs (mono-, di-, tri-, tetra-, penta-, and hexanucleotide repeats) found in the plastid genome of nine species, and the mononucleotide repeats were the most frequent (Figure 3(a), Table S1). The highest percentages of SSRs located in the LSC region were the maximum (69.44%-79.17%), and 4%-10% SSRs were distributed in IR regions (Figure 3(b), Table S2). A total of 41 types were detected in nine plastid genomes (Figure 3(c)), where bases T and A were the dominant elements. In this study, we manually chose 7 mononucleotide SSRs located in *trn*K-*rps*16, *psb*K-I, *acc*D-*psa*I, and *psa*J-*rpl*33 regions and *atp*F, *rpo*C1, and *pet*B genes as effective polymorphic SSRs between *Tulipa* species based on three critical criteria outlined in previous research [40]. These 7 mononucleotide SSRs could be used in the further population studies of *Tulipa* (Table S3).

### 3.3. Codon Usage Bias and SNP Analyses

**Figure 4:**
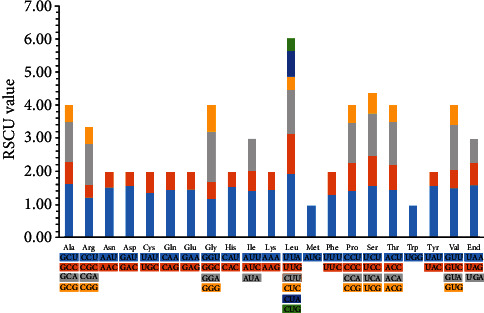
Codon content of 20 amino acids and stop codons in 84 coding genes of nine tribe Tulipeae plastid genomes. The color of the histogram corresponds to the color of codons.

The results of codon usage frequency and relative synonymous codon usage (RSCU) from 84 protein-coding sequences from nine tribe Tulipeae species are presented in Figure 4 and Table S4. The 84 protein-coding sequences were similar across the nine species. The total codon number from the nine species plastid genomes ranged between 21170 (*Erythronium sibiricum*) and 21284 (*T. thianschanica*) (Table S4). Among all amino acids, leucine and cysteine were the most and the least frequent, on average, 2165 (10.19%) and 333 (1.57%), respectively (Figure 4 and Table S4). The third codon position occupied by the A or T base was the most common in all nine tribe Tulipeae species, which is also found in other plastid genomes in seed plants [68–71]. Codon usage bias was related to gene expression and differed between species [72, 73]. Our results of codon usage bias will be important for understanding the molecular evolution mechanisms of *Tulipa* and its relatives.

**Figure 5:**
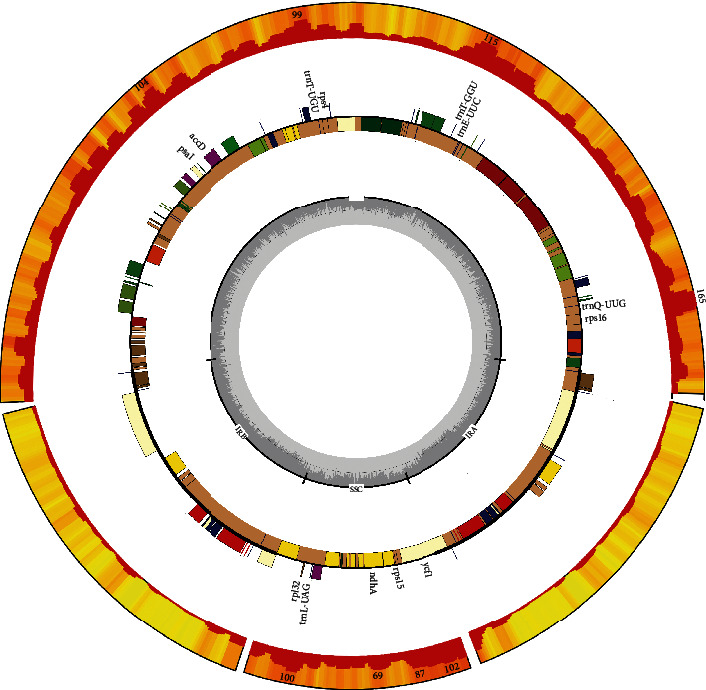
Statistics of the SNPs in the nine tribe Tulipeae plastid genomes. Red bars and heat map in the outer ring represent the SNP number. The locations and SNP numbers of eight highly variable regions (see text) are labeled.

The single-nucleotide polymorphism (SNP) analyses of the alignment for the nine tribe Tulipeae plastid genomes showed that the IR regions were more conserved than the LSC and SSC regions, where the SNP number was low (Figure 5), and was in agreement with previous reports for the angiosperm plastid genome [74, 75]. The noncoding regions were more variable than the coding-protein regions. In the sequence alignment of the nine tribe Tulipeae plastid genomes, six noncoding regions, *rps*16-*trn*Q, *trn*E-*trn*T, *acc*D-*psa*I, *rpl*32-*trn*L, *rps*15-*ycf*1, and *rps*4-*trn*T, and *ycf*1 and *ndh*A genes were highly variable.

### 3.4. Phylogenetic Analysis

**Figure 6:**
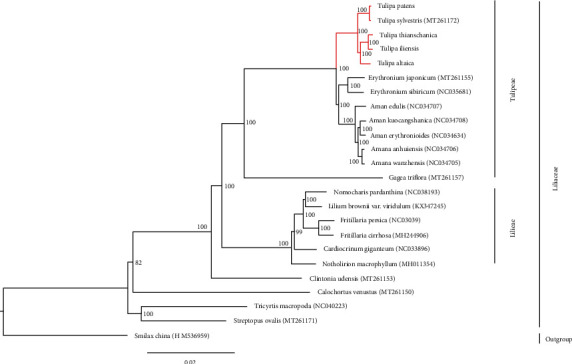
ML phylogenetic tree reconstruction containing the plastid genomes of 24 plants. *Smilax china* was set as the outgroup.

The phylogenetic relationship of *Tulipa* and other Liliaceae species was reconstructed based on 24 plastid genomes, representing 14 genera (Figure 6). The maximum likelihood (ML) tree strongly supported *Tulipa* as a monophyletic genus that was sister to *Amana* and *Erythronium* (1/100%), which was the same as previous research [29, 40]. *Tulipa*, *Amana*, *Erythronium*, and *Gagea* formed a monophyletic clade (1/100%). The ML analytical result of *Tulipa* and other Liliaceae species based on plastid genomes was in accordance with APG IV [17]. Previous studies could not resolve the phylogenetic relationships of *Tulipa*, *Amana*, and *Erythronium* because of insufficient data. In our ML tree, inferred from 23 Liliaceae species' plastid genomes, *Amana* and *Erythronium* clustered together with strong support (1/100%), and *Tulipa* was a sister to the *Amana*/*Erythronium* clade. The phylogenomics reconstructed distinct relationships among *Tulipa* and other genera.

In *Tulipa*, *T. iliensis* and *T. thianschanica* were very close and were sisters to *T. altaica*. We also found that *T. patens* was sister to *T. sylvestris*. In previous research [26, 38], *Tulipa* was divided into four subgenera: *Clusianae*, *Tulipa*, *Eriostemones*, and *Orithyia*. *T. iliensis* and *T. altaica* belong to subgenus *Tulipa* [37], yet *T. iliensis*, *T. thianschanica*, and *T. altaica* have similar morphological characteristics, and our results confirmed this grouping. *T. patens* and *T. sylvestris* were within the subgenus *Eriostemones*, and *T. patens* was once treated as a varietas of *T. sylvestris* [37]. Our phylogenetic tree based on plastid genomes demonstrated clear relationships between the five *Tulipa*, which was in accordance with a previous classification [37], and found that *T. thianschanica* should belong to subgenus *Tulipa*. Our results provide a better understanding of the evolution and molecular biology of *Tulipa*.

## 4. Conclusions

In this study, the plastid genome sequences of four*Tulipa* species were reported. We described the comparative characteristics of nine tribe Tulipeae plastid genomes and the phylogenetic relationships of 23 Liliaceae plastid genomes. We found that *Tulipa* plastid genomes were highly similar in overall size, structure, IR/SC boundary, SSRs, and codon usage bias. The phylogenetic tree identified a clear sister relationship between *Tulipa* and *Amana*, *Erythronium* and clear relationships between the five *Tulipa* species. Our study supplements the molecular data of *Tulipa* and provides a better understanding of *Tulipa* plastid genome evolution.

## Figures and Tables

**Table 1:** Genome features of five *Tulipa* plastid genomes.

Region	*T. altaica*	*T. iliensis*	*T. patens*	*T. thianschanica*	*T. sylvestris*
GenBank numbers	MW077741	MW077740	MW077739	MW077738	MT261172
Genome size (bp)	151,691	152,073	152,088	152,087	151,940
LSC (bp)	81,806	82,252	82,262	82,317	81,958
SSC (bp)	17,213	17,175	17,204	17,156	17,524
IR (bp)	26,336	26,323	26,311	26,307	26,341
Number of total genes	134	134	134	134	134
Protein-coding genes	79	79	79	79	79
tRNAs	30	30	30	30	30
rRNAs	4	4	4	4	4
Total GC content (%)	36.7	36.6	36.6	36.6	36.7
LSC GC content (%)	34.6	34.6	34.6	34.5	34.6
SSC GC content (%)	30.0	30.1	30.1	30.1	30.2
IR GC content (%)	42.0	42.0	42.0	42.0	42.0

**Table 2:** List of genes encoded in five *Tulipa* species.

Category for genes	Group of genes	Name of genes
Self-replication	Transfer RNAs	*trnA-UGC* ^∗^, *trnC-GCA*, *trnD-GUC*, *trnE-UUC*, *trnF-GAA*, *trnfM-CAU*, *trnG-GCC*, *trnG-UCC*, *trnH-GUG*^∗^, *trnI-CAU*^∗^, *trnI-GAU*^∗^, *trnK-UUU*, *trnL-CAA*^∗^, *trnL-UAA*, *trnL-UAG*, *trnM-CAU*, *trnN-GUU*^∗^, *trnP-UGG*, *trnQ-UUG*, *trnR-ACG*^∗^, *trnR-UCU*, *trnS-GCU*, *trnS-GGA*, *trnS-UGA*, *trnT-GGU*, *trnT-UGU*, *trnV-GAC*^∗^, *trnV-UAC*, *trnW-CCA*, *trnY-GUA*
	Ribosomal RNAs	*rrn4.5* ^∗^, *rrna5*^∗^, *rrn16*^∗^, *rrn23*^∗^
	RNA polymerase	*rpoA*, *rpoB*, *rpoC1*, *rpoC2*
	Small subunit of ribosomal proteins (SSU)	*rps2*, *rps3*, *rps4*, *rps7*^∗^, *rps8*, *rps11*, *rps12*^∗^, *rps14*, *rps15*, *rps16*, *rps18*, *rps19*^∗^ (*rps19*, *ψrps19*)
	Large subunit of ribosomal proteins (LSU)	*rpl2* ^∗^, *rpl14*, *rpl16*, *rpl20*, *rpl22*, *rpl23*^∗^, *rpl32*, *rpl33*, *rpl36*

Genes for photosynthesis	Subunits of NADH-dehydrogenase	*ndhA*, *ndhB*^∗^, *ndhC*, *ndhD*, *ndhE*, *ndhF*, *ndhG*, *ndhH*, *ndhI*, *ndhJ*, *ndhK*
	Subunits of photosystem I	*psaA*, *psaB*, *psaC*, *psaI*, *psaJ*
	Subunits of photosystem II	*psbA*, *psbB*, *psbC*, *psbD*, *psbE*, *psbF*, *psbH*, *psbI*, *psbJ*, *psbK*, *psbL*, *psbM*, *psbN*, *psbT*, *psbZ*
	Subunits of cytochrome b/f complex	*petA*, *petB*, *petD*, *petG*, *petL*, *petN*
	Subunits of ATP synthase	*atpA*, *atpB*, *atpE*, *atpF*, *atpH*, *atpI*
	Large subunit of rubisco	*rbcL*

Other genes	Protease	*clpP*
	Maturase	*matK*
	Subunit of acetyl-CoA-carboxylase	*accD*
	Envelope membrane protein	*cemA*
	C-type cytochrome synthesis gene	*ccsA*

Genes of unknown function	Hypothetical chloroplast reading frames	*ycf1* ^∗^ (*ycf1*, *ψycf1*), *ycf2*^∗^, *ycf3*, *ycf4*, *ψycf68*^∗^

^∗^Duplicated genes; *ψ* shows pseudogenes.

## Data Availability

The assembled plastid genome sequences of the four species were submitted to NCBI with the accession numbers MW077741 (*T. altaica*), MW077740 (*T. iliensis*), MW077739 (*T. patens*), and MW077738 (*T. thianschanica*). Users can download the data as a reference for research purposes only.

## Supplementary Materials

Supplementary Materials Table S1: number of different SSR categories detected in nine species. Table S2: the frequency of identified SSRs in LSC, IR, and SSC of nine species. Table S3: seven polymorphic SSRs between *Tulipa* species. Table S4: the codon numbers of amino acids in nine plastid genomes.

## References

[1] Wicke S., Schneeweiss G. M., dePamphilis C. W., Müller K. F., Quandt D. (2011). The evolution of the plastid chromosome in land plants: gene content, gene order, gene function. *Plant Molecular Biology*.

[2] Daniell H., Lin C. S., Yu M., Chang W. J. (2016). Chloroplast genomes: diversity, evolution, and applications in genetic engineering. *Genome Biology*.

[3] Park M., Park H., Lee H., Lee B.-h., Lee J. (2018). The Complete Plastome Sequence of an Antarctic Bryophyte Sanionia uncinata (Hedw.) Loeske. *International Journal of Molecular Sciences*.

[4] Dong W. P., Liu H., Xu C., Zuo Y., Chen Z., Zhou S. (2014). A chloroplast genomic strategy for designing taxon specific DNA mini-barcodes: a case study on ginsengs. *BMC Genetics*.

[5] Nadachowska-Brzyska K., Li C., Smeds L., Zhang G., Ellegren H. (2015). Temporal dynamics of avian populations during Pleistocene revealed by whole-genome sequences. *Current Biology*.

[6] Xie D. F., Yu Y., Deng Y. Q. (2018). Comparative analysis of the chloroplast genomes of the Chinese endemic genus Urophysa and their contribution to chloroplast phylogeny and adaptive evolution. *International Journal of Molecular Sciences*.

[7] Liu H. Y., Yu Y., Deng Y. Q., Li J., Huang Z.-X., Zhou S.-D. (2018). The chloroplast genome of Lilium henrici: genome structure and comparative analysis. *Molecules*.

[8] Jansen R. K., Raubeson L. A., Boore J. L. (2005). Methods for obtaining and analyzing whole chloroplast genome sequences. *Methods in Enzymology*.

[9] Jansen R. K., Ruhlman T. A., Knoop R. V. (2012). Plastid genomes of seed plants. *Advances in Photosynthesis and Respiration vol. 35*.

[10] Guo X. L., Zheng H. Y., Megan P., Zhou S.-D., He X.-J. (2020). Phylogeny and comparative analysis of Chinese Chamaesium species revealed by the complete plastid genome. *Plants*.

[11] Dong W., Liu J., Yu J., Wang L., Zhou S. (2012). Highly variable chloroplast markers for evaluating plant phylogeny at low taxonomic levels and for DNA barcoding. *PLoS One*.

[12] Ruben S., Carlos P. C., Diana L. A. (2017). Comparative plastome genomics and phylogenomics of Brachypodium: flowering time signatures, introgression and recombination in recently diverged ecotypes. *New Phytologist*.

[13] Hong T. L., Yi T. S., Gao L. M. (2019). Origin of angiosperms and the puzzle of the Jurassic gap. *Nature plants*.

[14] Van C. M., Kerckhoffs D., Van T. J. (1997). Interspecific crosses in the genus Tulipa L.: identification of pre-fertilization barriers. *Sexual Plant Reprodection*.

[15] Juodkaite R., Baliuniene A., Naujalis J. R., Navalinskienė M., Samuitienė M. (2008). Selection and presentation of tulip (Tulipa L.) species and cultivars to the Lithuanian plant genetic resources. *Biologia*.

[16] Van S. J. (1996). Classified list and international register of plant genetic resources. *Biologia*.

[17] The Angiosperm Phylogeny Group (2016). Angiosperm Phylogeny Group: An update of the Angiosperm Phylogeny Group classification for the orders and families of flowering plants: APG IV. *Botanical Journal of the Linnean Society*.

[18] Tamura M. N., Kubitzki K. (1998). Liliaceae. *Flowering Plants Monocotyledons vol. 3*.

[19] Wendelbo P., Rechinger K. H., Rechinger K. H. (1990). *Gagea, Flora Iranica vol. 165*.

[20] Angela P., Orte H., Jens P., Alexander H., Lorenzo P. (2019). A pre‐Miocene Irano‐Turanian cradle: Origin and diversification of the species‐rich monocot genus Gagea (Liliaceae). *Ecology and Evolution*.

[21] Kim J. S., Kim J. H. (2018). Updated molecular phylogenetic analysis, dating and biogeographical history of the lily family (Liliaceae: Liliales). *Botanical Journal of the Linnean Society*.

[22] Allen G. A., Soltis D. E., Soltis P. S. (2003). Phylogeny and biogeography of Erythronium (Liliaceae) inferred from chloroplast matK and nuclear rDNA ITS sequences. *Systematic Botany*.

[23] Petersen G., Seberg O., Davis J. I. (2013). Phylogeny of the Liliales (monocotyledons) with special emphasis on data partition congruence and RNA editing. *Cladistics*.

[24] Eijk J. V., Raamsdonk L. V., Eikelboom W., Bino R. J. (1991). Interspecific crosses between Tulipa gesneriana cultivars and wild Tulipa species: a survey. *Sexual Plant Reprodection*.

[25] van Rossum M. W., Alberda M., van der Plas L. H. (1998). Tulipaline and tuliposide in cultured explants of tulip bulb scales. *Phytochemistry*.

[26] Zonneveld B. J. (2009). The systematic value of nuclear genome size for “all” species of Tulipa L. (Liliaceae). *Plant Systematics and Evolution*.

[27] Tan D. Y., Li X. R., Hong D. Y. (2008). Neotypification and additional description of Amana anhuiensis (X.S.Shen) D.Y.Tan & D.Y.Hong (Liliaceae) from Anhui,China. *Acta Botanica Boreali-Occidentalia Sinica*.

[28] Han B. X., Zhang K., Huang L. Q. (2014). Amana wanzhensis (Liliaceae), a new species from Anhui, China. *Phytotaxa*.

[29] Christenhusz M. J. M., Govaerts R., David J. C. (2013). Tiptoe through the tulips - cultural history, molecular phylogenetics and classification of Tulipa (Liliaceae). *Botanical Journal of the Linnean Society*.

[30] Hayashi K., Kawano H. (2000). Molecular systematics of Lilium and allied genera (Liliaceae): phylogenetic relationships among Lilium and related genera based on the rbcL and matK gene sequence data. *Plant Species Biology*.

[31] Zarrei M., Wilkin P., Fay M. F., Ingrouille M. J., Zarre S., Chase M. W. (2009). Molecular systematics of Gagea and Lloydia (Liliaceae Liliales): implications of analyses of nuclear ribosomal and plastid DNA sequences for infrageneric classification. *Annals of Botany*.

[32] Rønsted N., Law S., Thornton H., Fay M. F., Chase M. W. (2005). Molecular phylogenetic evidence for the monophyly of Fritillaria and Lilium (Liliaceae; Liliales) and the infrageneric classification of Fritillaria. *Molecular Phylogenetics and Evolution*.

[33] Kim J. S., Kim J. H. (2013). Comparative genome analysis and phylogenetic relationship of order Liliales insight from the complete plastid genome sequences of two Lilies (Lilium longiflorumand, Alstroemeria aurea). *PLoS One*.

[34] Hall A. D., Smith W. W. (1940). *The genus Tulipa*.

[35] Marasek A., Okazaki K. (2008). Analysis of introgression of the Tulipa fosteriana genome into Tulipa gesneriana using GISH and FISH. *Euphytica*.

[36] Botschantzeva Z. P., Samuel B. J. (1982). *Tulips: taxonomy, morphology, cytology, phytogeography, and physiology*.

[37] Chen X., Helen V. M., Samuel B. J., Wu Z. Y., Raven P. H. (2000). *Tulipa Linnaeus, Flora of China*.

[38] Mine T., Ozge K., Metin B. B. T. (2013). Molecular phylogenetic analysis of Tulipa (Liliaceae) based on noncoding plastid and nuclear DNA sequences with an emphasis on Turkey. *Botanical Journal of the Linnean Society*.

[39] Davoud A., Alireza B., Mohammad R. N., Mahmoud K. (2020). Biodiversity status of Tulipa (Liliaceae) in Iran inferred from molecular characterization. *Horticulture, Environment, and Biotechnology*.

[40] Li P., Lu R. S., Xu W. Q. (2017). Comparative genomics and phylogenomics of east Asian Tulips (Amana, Liliaceae). *Frontiers in Plant Science*.

[41] Do H. D. K., Kim C., Chase M. W., Kim J. H. (2020). Implications of plastome evolution in the true lilies (monocot order Liliales). *Molecular Phylogenetics and Evolution*.

[42] Yang J. B., Li D. Z., Li H. T. (2014). Highly effective sequencing whole chloroplast genomes of angiosperms by nine novel universal primer pairs. *Molecular Ecology Resources*.

[43] Dierckxsens N., Mardulyn P., Smits G. (2017). NOVOPlasty: de novo assembly of organelle genomes from whole genome data. *Nucleic Acids Research*.

[44] Qu X. J., Moore M. J., Li D. Z., Yi T. S. (2019). PGA: a software package for rapid, accurate, and flexible batch annotation of plastomes. *Plant Methods*.

[45] Kearse M., Moir R., Wilson A. (2012). Geneious basic: an integrated and extendable desktop software platform for the organization and analysis of sequence data. *Bioinformatics*.

[46] Lohse M., Drechsel O., Kahlau S., Bock R. (2013). OrganellarGenomeDRAW—a suite of tools for generating physical maps of plastid and mitochondrial genomes and visualizing expression data sets. *Nucleic Acids Research*.

[47] Amiryousefi A., Hyvönen J., Poczai P. (2018). Irscope: an online program to visualize the junction sites of chloroplast genomes. *Bioinformatics*.

[48] Thiel T., Michalek W., Varshney R. K., Graner A. (2003). Exploiting EST databases for the development and characterization of gene-derived SSR-markers in barley (Hordeum vulgare L.). *Theoretical and Applied Genetics*.

[49] Katoh K., Standley D. M. (2013). MAFFT multiple sequence alignment software version 7: improvements in performance and usability. *Molecular Biology and Evolution*.

[50] Stamatakis A. (2006). RAxML-VI-HPC: maximum likelihood-based phylogenetic analyses with thousands of taxa and mixed models. *Bioinformatics*.

[51] Librado P., Rozas J. (2009). DnaSP v5: a software for comprehensive analysis of DNA polymorphism data. *Bioinformatics*.

[52] Chen C. J., Chen H., Zhang Y. (2020). TBtools: an integrative toolkit developed for interactive analyses of big biological data. *Molecular Plant*.

[53] Lu R. S., Li P., Qiu Y. X. (2017). The complete chloroplast genomes of three Cardiocrinum (Liliaceae) species: comparative genomic and phylogenetic analyses. *Frontiers in Plant Science*.

[54] Raubeson L. A., Peery R., Chumley T. W. (2007). Comparative chloroplast genomics: analyses including new sequences from the angiosperms Nuphar advena and Ranunculus macranthus. *BMC Genomics*.

[55] Wang R. J., Cheng C. L., Chang C. C., Wu C.-L., Su T.-M., Chaw S.-M. (2008). Dynamics and evolution of the inverted repeat-large single copy junctions in the chloroplast genomes of monocots. *BMC Evolutionary Biology*.

[56] Yang M., Zhang X., Liu G. (2010). The complete chloroplast genome sequence of date palm (Phoenix dactylifera L.). *PLoS One*.

[57] Kim K. J., Lee H. L. (2004). Complete chloroplast genome sequences from Korean ginseng (Panax schinseng Nees) and comparative analysis of sequence evolution among 17 vascular plants. *DNA Research*.

[58] Parks M., Cronn R., Liston A. (2009). Increasing phylogenetic resolution at low taxonomic levels using massively parallel sequencing of chloroplast genomes. *BMC Biology*.

[59] Wheeler G. L., Dorman H. E., Buchanan A. T., Challagundla L., Wallace L. E. (2014). A review of the prevalence, utility, and caveats of using chloroplast simple sequence repeats for studies of plant biology. *Applications in Plant Sciences*.

[60] Ebert D., Peakall R. (2009). Chloroplast simple sequence repeats (cpSSRs): technical resources and recommendations for expanding cpSSR discovery and applications to a wide array of plant species. *Molecular Ecology Resources*.

[61] Yang A., Zhang J., Yao X., Huang H. (2011). Chloroplast microsatellite markers in Liriodendron tulipifera (Magnoliaceae) and cross-species amplification in L. chinense. *American Journal of Botany*.

[62] Dong W., Xu C., Li D. (2016). Comparative analysis of the complete chloroplast genome sequences in psammophytic Haloxylon species (Amaranthaceae). *Peer J*.

[63] Kaur S., Panesar P. S., Bera M. B., Kaur V. (2015). Simple sequence repeat markers in genetic divergence and marker-assisted selection of rice cultivars: a review. *Critical Reviews in Food ence & Nutrition*.

[64] Yang Y., Zhou T., Duan D., Yang J., Feng L., Zhao G. (2016). Comparative analysis of the complete chloroplast genomes of five Quercus species. *Frontiers in Plant Science*.

[65] Powell W., Morgante M., McDevitt R., Vendramin G. G., Rafalski J. A. (1995). Polymorphic simple sequence repeat regions in chloroplast genomes-applications to the population genetics of pines. *Proceedings of the National Academy of Sciences of the United States of America*.

[66] Provan J., Corbett G., McNicol J. W., Powell W. (1997). Chloroplast DNA variability in wild and cultivated rice (Oryza spp.) revealed by polymorphic chloroplast simple sequence repeats. *Genome*.

[67] Pauwels M., Vekemans X., Gode C., Frérot H., Castric V., Saumitou‐Laprade P. (2012). Nuclear and chloroplast DNA phylogeography reveals vicariance among European populations of the model species for the study of metal tolerance, Arabidopsis halleri (Brassicaceae). *New Phytologist*.

[68] Clegg M. T., Gaut B. S., Learn G. H., Morton B. R. (1994). Rates and patterns of chloroplast DNA evolution. *Proceedings of the National Academy of Sciences*.

[69] Liu Q., Xue Q. (2004). Codon usage in the chloroplast genome of rice (Oryza sativa L. ssp. japonica). *Acta Agronomica Sinica*.

[70] Zhou M., Long W., Li X. (2008). Analysis of synonymous codon usage in chloroplast genome of Populus alba. *Journal of Forestry Research*.

[71] Tangphatsornruang S., Sangsrakru D., Chanprasert J. (2010). The chloroplast genome sequence of mungbean (vigna radiata) determined by high-throughput pyrosequencing: structural organization and phylogenetic relationships. *DNA Research*.

[72] Du Y., Bi Y., Chen X., Yang F., Xue J., Zhang X. (2016). The complete chloroplast genome of Lilium cernuum: genome structure and evolution. *Conservation Genetics Resources*.

[73] Morton B. R. (1998). Selection on the codon bias of chloroplast and cyanelle genes in different plant and algal lineages. *Journal of Molecular Evolution*.

[74] Meng J., Li X., Li H. T., Yang J., Wang H., He J. (2018). Comparative analysis of the complete chloroplast genomes of four Aconitum medicinal species. *Molecules*.

[75] Liang C. L., Wang L., Le J. (2019). A comparative analysis of the chloroplast genomes of four Salvia medicinal plants. *Engineering*.

