# Supplementary material for: Phylogeny and Comparative Analysis for the Plastid Genomes of Five Tulipa (Liliaceae)
Source: Biomed Res Int. 2021 Jun 18;2021:6648429. doi: 10.1155/2021/6648429 (PMC8235973; doi:10.1155/2021/6648429)
Supplement: Supplementary Materials — Table S1: number of different SSR categories detected in nine species. Table S2: the frequency of identified SSRs in LSC, IR, and SSC of nine species. Table S3: seven polymorphic SSRs between Tulipa species. Table S4: the codon numbers of amino acids in nine plastid genomes. [file 6648429.f1.zip › TableS1.docx]

**Table S1.** Number of different SSRs categories detected in nine species.

| **Taxonomy** | **Mono-** | **Di-** | **Tri-** | **Tetra-** | **Penta-** | **Hexa-** | **Tatal** |
| --- | --- | --- | --- | --- | --- | --- | --- |
| *T. thianschanica* | 42 | 19 | 2 | 8 | 1 | 0 | 72 |
| *T. patens* | 45 | 19 | 3 | 9 | 1 | 1 | 78 |
| *T. iliensis* | 47 | 18 | 3 | 7 | 1 | 1 | 77 |
| *T. altaica* | 42 | 17 | 3 | 7 | 0 | 3 | 72 |
| *T. sylvestris* | 49 | 18 | 3 | 8 | 1 | 1 | 80 |
| *G. triflora* | 34 | 14 | 4 | 12 | 0 | 0 | 64 |
| *E. sibiricum* | 38 | 25 | 6 | 10 | 4 | 0 | 83 |
| *E. japonicum* | 43 | 21 | 6 | 7 | 2 | 0 | 79 |
| *A. edulis* | 35 | 20 | 5 | 10 | 1 | 0 | 71 |
