# Supplementary material for: Phylogeny and Comparative Analysis for the Plastid Genomes of Five Tulipa (Liliaceae)
Source: Biomed Res Int. 2021 Jun 18;2021:6648429. doi: 10.1155/2021/6648429 (PMC8235973; doi:10.1155/2021/6648429)
Supplement: Supplementary Materials — Table S1: number of different SSR categories detected in nine species. Table S2: the frequency of identified SSRs in LSC, IR, and SSC of nine species. Table S3: seven polymorphic SSRs between Tulipa species. Table S4: the codon numbers of amino acids in nine plastid genomes. [file 6648429.f1.zip › TableS2.docx]

**Table S2.** The frequency of identified SSRs in LSC, IR, SSC of nine species.

| **Taxonomy** | **LSC** | **IR** | **SSC** | **Total** |
| --- | --- | --- | --- | --- |
| ***T. thianschanica*** | 57 | 4 | 11 | 72 |
| ***T. patens*** | 61 | 4 | 13 | 78 |
| ***T. iliensis*** | 58 | 6 | 13 | 77 |
| ***T. altaica*** | 50 | 8 | 14 | 72 |
| ***T. sylvestris*** | 62 | 6 | 12 | 80 |
| ***G. triflora*** | 46 | 10 | 8 | 64 |
| ***E. sibiricum*** | 62 | 6 | 15 | 83 |
| ***E. japonicum*** | 57 | 6 | 16 | 79 |
| ***A. edulis*** | 53 | 6 | 12 | 71 |
