# Supplementary material for: Phylogeny and Comparative Analysis for the Plastid Genomes of Five Tulipa (Liliaceae)
Source: Biomed Res Int. 2021 Jun 18;2021:6648429. doi: 10.1155/2021/6648429 (PMC8235973; doi:10.1155/2021/6648429)
Supplement: Supplementary Materials — Table S1: number of different SSR categories detected in nine species. Table S2: the frequency of identified SSRs in LSC, IR, and SSC of nine species. Table S3: seven polymorphic SSRs between Tulipa species. Table S4: the codon numbers of amino acids in nine plastid genomes. [file 6648429.f1.zip › TableS3.docx]

Table S3. Seven effective polymorphic SSRs between *Tulipa* species.

| **Taxonomy** | ***T. thianschanica*** | ***T. patens*** | ***T. iliensis*** | ***T. altaica*** | ***T. sylvestris*** |
| --- | --- | --- | --- | --- | --- |
| trnK-UUU-rps16 (A) | | | | | |
| repeats | 15 | 10 | 13 | 13 | 10 |
| location | 4152-4166 | 4124-4143 | 4159-4171 | 4147-4159 | 4114-4123 |
| psbK-psbI (A) | | | | | |
| repeats | 11 | 17 | 15 | 14 | 17 |
| location | 7531-7541 | 7533-7549 | 7525-7539 | 7573-7586 | 7508-7524 |
| atpF (A) | | | | | |
| repeats | 15 | 25 | 11 | 16 | 22 |
| location | 12493-12507 | 12529-12553 | 12510-12520 | 12579-12594 | 12521-12542 |
| rpoC1 (T) | | | | | |
| repeats | 11 | 12 | 20 | 10 | 11 |
| location | 22030-22040 | 22074-22085 | 22044-22063 | 22067-22076 | 22067-22077 |
| accD-psaI (A) | | | | | |
| repeats | 20 | 15 | 20 | 14 | 32 |
| location | 58182-58201 | 57989-58003 | 58142-58161 | 58143-58156 | 57837-57868 |
| psaJ-rpl33 (A) | | | | | |
| repeats | 14 | 21 | 14 | 16 | 15 |
| location | 66083-66096 | 65944-65964 | 65971-65984 | 65606-65621 | 65756-65770 |
| petB (A) | | | | | |
| repeats | 11 | 15 | 12 | 12 | 12 |
| location | 74034-74044 | 73915-73929 | 73939-73950 | 73515-73526 | 73733-73744 |
