# Supplementary material for: Phylogeny and Comparative Analysis for the Plastid Genomes of Five Tulipa (Liliaceae)
Source: Biomed Res Int. 2021 Jun 18;2021:6648429. doi: 10.1155/2021/6648429 (PMC8235973; doi:10.1155/2021/6648429)
Supplement: Supplementary Materials — Table S1: number of different SSR categories detected in nine species. Table S2: the frequency of identified SSRs in LSC, IR, and SSC of nine species. Table S3: seven polymorphic SSRs between Tulipa species. Table S4: the codon numbers of amino acids in nine plastid genomes. [file 6648429.f1.zip › TableS4.docx]

**Table S4.** The codon numbers of amino acid in nine plastid genomes.

| amino acid | codon | *Amana edulis* | *Erythronium japonicum* | *Erythronium sibiricum* | *Gagea triflora* | *Tulipa altaica* | *Tulipa iliensis* | *Tulipa patens* | *Tulipa sylvestris* | *Tulipa thianschanica* | total number |
| --- | --- | --- | --- | --- | --- | --- | --- | --- | --- | --- | --- |
| Phe | UUU | 1029 | 804 | 795 | 795 | 792 | 795 | 796 | 795 | 799 | 7400 |
|  | UUC | 512 | 543 | 390 | 389 | 398 | 400 | 390 | 392 | 400 | 3814 |
| Leu | UUA | 413 | 527 | 753 | 772 | 762 | 760 | 768 | 768 | 759 | 6282 |
|  | UUG | 319 | 629 | 427 | 425 | 424 | 426 | 421 | 422 | 427 | 3920 |
|  | CUU | 347 | 460 | 481 | 476 | 488 | 487 | 485 | 486 | 488 | 4198 |
|  | CUC | 123 | 317 | 127 | 128 | 124 | 125 | 125 | 127 | 125 | 1321 |
|  | CUA | 149 | 411 | 271 | 282 | 276 | 277 | 276 | 272 | 276 | 2490 |
|  | CUG | 90 | 335 | 121 | 117 | 122 | 121 | 122 | 123 | 121 | 1272 |
| Ile | AUU | 741 | 673 | 900 | 910 | 907 | 906 | 906 | 902 | 906 | 7751 |
|  | AUC | 362 | 425 | 343 | 328 | 342 | 348 | 344 | 346 | 348 | 3186 |
|  | AUA | 339 | 623 | 627 | 610 | 629 | 622 | 630 | 628 | 624 | 5332 |
| Met | AUG | 231 | 527 | 493 | 505 | 498 | 504 | 498 | 499 | 503 | 4258 |
| Val | GUU | 249 | 340 | 444 | 430 | 444 | 445 | 444 | 444 | 443 | 3683 |
|  | GUC | 127 | 199 | 143 | 147 | 147 | 144 | 145 | 145 | 145 | 1342 |
|  | GUA | 99 | 304 | 433 | 440 | 435 | 434 | 433 | 435 | 433 | 3446 |
|  | GUG | 89 | 252 | 156 | 150 | 150 | 151 | 155 | 155 | 150 | 1408 |
| Ser | UCU | 460 | 334 | 450 | 451 | 456 | 459 | 453 | 455 | 461 | 3979 |
|  | UCC | 363 | 237 | 234 | 251 | 233 | 232 | 232 | 232 | 228 | 2242 |
|  | UCA | 552 | 382 | 345 | 321 | 340 | 339 | 335 | 334 | 341 | 3289 |
|  | UCG | 325 | 277 | 133 | 149 | 140 | 141 | 142 | 142 | 139 | 1588 |
| Pro | CCU | 170 | 172 | 338 | 327 | 337 | 333 | 339 | 339 | 334 | 2689 |
|  | CCC | 136 | 145 | 184 | 197 | 184 | 187 | 184 | 183 | 186 | 1586 |
|  | CCA | 199 | 244 | 247 | 256 | 250 | 252 | 255 | 255 | 251 | 2209 |
|  | CCG | 100 | 181 | 97 | 97 | 100 | 96 | 96 | 96 | 98 | 961 |
| Thr | ACU | 255 | 187 | 437 | 432 | 433 | 434 | 433 | 433 | 433 | 3477 |
|  | ACC | 258 | 181 | 180 | 186 | 181 | 182 | 183 | 183 | 182 | 1716 |
|  | ACA | 370 | 275 | 341 | 341 | 342 | 342 | 337 | 337 | 340 | 3025 |
|  | ACG | 203 | 173 | 109 | 111 | 110 | 107 | 112 | 113 | 109 | 1147 |
| Ala | GCU | 135 | 104 | 521 | 542 | 530 | 529 | 533 | 533 | 531 | 3958 |
|  | GCC | 96 | 85 | 183 | 172 | 179 | 179 | 179 | 179 | 179 | 1431 |
|  | GCA | 147 | 125 | 348 | 341 | 353 | 349 | 348 | 348 | 352 | 2711 |
|  | GCG | 85 | 117 | 115 | 126 | 116 | 118 | 120 | 120 | 116 | 1033 |
| Tyr | UAU | 853 | 647 | 675 | 672 | 677 | 679 | 674 | 673 | 675 | 6225 |
|  | UAC | 376 | 334 | 142 | 148 | 149 | 149 | 152 | 152 | 149 | 1751 |
| His | CAU | 341 | 300 | 415 | 410 | 420 | 416 | 416 | 416 | 416 | 3550 |
|  | CAC | 139 | 160 | 104 | 108 | 99 | 98 | 101 | 100 | 99 | 1008 |
| Gln | CAA | 344 | 437 | 568 | 574 | 573 | 570 | 572 | 572 | 568 | 4778 |
|  | CAG | 135 | 303 | 183 | 187 | 182 | 185 | 184 | 184 | 185 | 1728 |
| Asn | AAU | 894 | 696 | 830 | 804 | 831 | 828 | 835 | 832 | 834 | 7384 |
|  | AAC | 361 | 343 | 218 | 222 | 219 | 219 | 220 | 220 | 218 | 2240 |
| Lys | AAA | 790 | 828 | 848 | 825 | 853 | 863 | 855 | 849 | 861 | 7572 |
|  | AAG | 342 | 555 | 267 | 272 | 268 | 265 | 265 | 264 | 268 | 2766 |
| Asp | GAU | 420 | 407 | 689 | 691 | 694 | 696 | 695 | 694 | 697 | 5683 |
|  | GAC | 160 | 179 | 165 | 170 | 164 | 166 | 164 | 164 | 167 | 1499 |
| Glu | GAA | 436 | 484 | 848 | 854 | 857 | 855 | 861 | 860 | 854 | 6909 |
|  | GAG | 196 | 331 | 258 | 262 | 264 | 263 | 263 | 263 | 264 | 2364 |
| Cys | UGU | 447 | 260 | 175 | 179 | 172 | 173 | 173 | 173 | 173 | 1925 |
|  | UGC | 418 | 197 | 63 | 65 | 66 | 65 | 65 | 64 | 65 | 1068 |
| Trp | UGG | 551 | 412 | 384 | 376 | 385 | 385 | 385 | 384 | 385 | 3647 |
| Arg | CGU | 143 | 97 | 295 | 293 | 298 | 299 | 297 | 298 | 298 | 2318 |
|  | CGC | 112 | 74 | 76 | 81 | 76 | 74 | 78 | 77 | 75 | 723 |
|  | CGA | 256 | 167 | 268 | 277 | 274 | 272 | 273 | 273 | 271 | 2331 |
|  | CGG | 169 | 176 | 91 | 93 | 85 | 84 | 84 | 84 | 85 | 951 |
| Ser | AGU | 403 | 210 | 348 | 346 | 347 | 345 | 344 | 344 | 343 | 3030 |
|  | AGC | 420 | 173 | 85 | 83 | 84 | 87 | 86 | 86 | 86 | 1190 |
| Arg | AGA | 609 | 319 | 399 | 390 | 401 | 399 | 401 | 400 | 399 | 3717 |
|  | AGG | 432 | 253 | 101 | 115 | 105 | 109 | 104 | 104 | 107 | 1430 |
| Gly | GGU | 182 | 187 | 476 | 470 | 477 | 478 | 478 | 477 | 478 | 3703 |
|  | GGC | 211 | 151 | 161 | 173 | 168 | 168 | 169 | 170 | 168 | 1539 |
|  | GGA | 359 | 275 | 572 | 569 | 573 | 574 | 574 | 575 | 574 | 4645 |
|  | GGG | 321 | 270 | 247 | 260 | 244 | 242 | 243 | 242 | 242 | 2311 |
| End | UAA | 539 | 478 | 31 | 30 | 31 | 31 | 30 | 29 | 32 | 1231 |
|  | UAG | 279 | 527 | 11 | 11 | 11 | 11 | 11 | 12 | 10 | 883 |
|  | UGA | 532 | 381 | 11 | 12 | 11 | 11 | 12 | 12 | 11 | 993 |
